# Childhood Obesity: A Potential Key Factor in the Development of Glioblastoma Multiforme

**DOI:** 10.3390/life12101673

**Published:** 2022-10-21

**Authors:** Punya Sachdeva, Shampa Ghosh, Soumya Ghosh, Sungsoo Han, Juni Banerjee, Rakesh Bhaskar, Jitendra Kumar Sinha

**Affiliations:** 1 GloNeuro, Sector 107, Vishwakarma Road, Noida 201301, India; 2 ICMR—National Institute of Nutrition, Tarnaka, Hyderabad 500007, India; 3 School of Chemical Engineering, Yeungnam University, Gyeongsan 38541, Korea; 4 Department of Biotechnology and Bioengineering, Institute of Advanced Research, Gandhinagar 382426, India

**Keywords:** adipocytes, adiponcosis, cancer, cytokines, inflammation, metabolic dysregulation, survival, tumorigenesis

## Abstract

Glioblastoma multiforme (GBM) is a malignant primary tumor type of the central nervous system (CNS). This type of brain tumor is rare and is responsible for 12–15% of all brain tumors. The typical survival rate of GBM is only 12 to 14 months. GBM has a poor and unsatisfactory prognosis despite advances in research and therapeutic interventions via neurosurgery, radiation, and chemotherapy. The molecular heterogeneity, aggressive nature, and occurrence of drug-resistant cancer stem cells in GB restricts the therapeutic efficacy. Interestingly, the CNS tumors in children are the second most usual and persistent type of solid tumor. Since numerous research studies has shown the association between obesity and cancer, childhood obesity is one of the potential reasons behind the development of CNS tumors, including GBM. Obesity in children has almost reached epidemic rates in both developed and developing countries, harming children’s physical and mental health. Obese children are more likely to face obesity as adults and develop non-communicable diseases such as diabetes and cardiovascular disease as compared to adults with normal weight. However, the actual origin and cause of obesity are difficult to be pointed out, as it is assumed to be a disorder with numerous causes such as environmental factors, lifestyle, and cultural background. In this narrative review article, we discuss the various molecular and genetic drivers of obesity that can be targeted as potential contributing factors to fight the development of GBM in children.

## 1. Introduction

In 1865, a German pathologist named Rudolf Virchow first reported the histomorphology of glial tumors arising from glial cells of the central nervous system (CNS) as malignant [1]. Harvey Cushing (1869–1939), also known as the father of modern neurosurgery, along with Percival Bailey (1892–1973), an American neuropathologist, classified gliomas as the most histologically atypical malignant tumors, and named them “spongioblastoma multiforme” due to the variations in the cells’ appearance within the tissues [2]. Other classified and discrete tumors, such as astrocytomas, originate from astrocytic glial cells. However, later the term “spongioblastoma” was replaced by “glioblastoma”, and the name “glioblastoma multiforme” (GBM) was established [3].

GBM has been reported as the most prevalent and intrinsic brain tumor originating from progenitor or neuroglial cells [4]. Some histological characteristics manifested by GBM are endothelial proliferation, amphophilic cytoplasm, hypercellularity, necrosis, prominent nucleoli, and nuclear atypia. The World Health Organization has assigned it one of the highest grades (grade IV) of tumor [5]. The peak age of its occurrence is 55–60 years [6], with a survival period of 14–15 months after diagnosis [6]. The GBM can be either primary when diagnosed as fully mature de novo tumors developed due to several genetic alterations, or secondary, progressing slowly from a low-grade astrocytoma [5]. On the other hand, primary glioblastomas are commonly seen in older people, and secondary glioblastomas frequently develop in younger ones [7,8]. Various computational tools have so far categorized GBM into four molecular subtypes: (i) classical (C); (ii) mesenchymal (M); (iii) proneural (P); (iv) neural (N). They can be distinguished based on their molecular profile signatures and prognosis, but not so much from their morphology [9]

## 2. Etiology of GBM

Brain neoplasms are usually incurable, and definite carcinogenic elements are yet to be discovered. To date, high levels of ionizing radiation exposure in the brain are one of the confirmed causes leading to the origin of brain tumors [10]. The first association between the brain tumors and ionizing radiation was observed in a study on Israeli children with tinea capitis who received radiation therapy [11]. Numerous studies on Japanese people to examine the consequences of radiation during the atomic explosion of Hiroshima and Nagasaki reported elevated chances for developing all types of tumors, including gliomas in children and adults [12]. Type 1 and type 2 neurofibromatosis and tuberous sclerosis are rare genetic disorders linked with a high incidence of glioma [13]. Another plausible cause is the effect of several drugs such as antihistamines and non-steroidal anti-inflammatory drugs (NSAIDs), which show an inverse link in the development of all gliomas [14]. Moreover, The Cancer Genome Atlas (TCGA) has identified alterations in the three pathways resulting in GBM formation: the retinoblastoma signaling, p53 signaling, and receptor tyrosine kinase signaling pathways [15]. Interestingly, several more probable risk factors, such as head injuries, cytomegalovirus infection, and body mass index (BMI), are correlated with the formation of neoplasms in the CNS [14].

## 3. Neuropsychiatric Manifestations of GBM

The patients suffering from GBM develop neurocognition impairment, leading to cognitive, behavioral, and emotional difficulties [16]. The symptoms depend on the location of the tumor in the cerebral cortex. Table 1 shows the location of the GBM tumor and the neuropsychiatric symptoms developed by the patients.

## 4. Obesity: A Cause of Cancer

**Figure 1:**
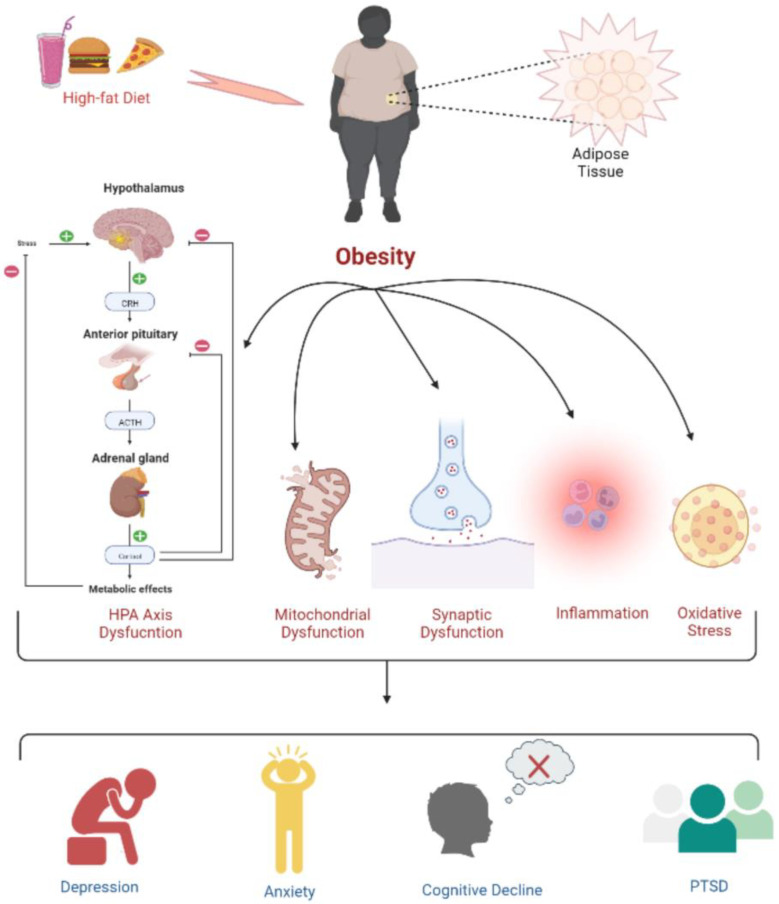
Psychophysiological changes due to high-fat-diet induced obesity. Obesity leads to a dysregulated HPA axis, mitochondrial and synaptic dysfunction, inflammation, and oxidative stress, which is further associated with the development of depression, anxiety, cognitive deficits, and PTSD.

Obesity and overweight are two prime risk factors for disorders related to hormones, blood circulation, and pulmonary functions, as they significantly contribute to chronic diseases such as atherosclerotic cardiovascular disease (ASCVD), type 2 diabetes, and asthma. Obesity causes inflammation, oxidative stress, and dysfunctions related to the hypothalamic–pituitary–adrenal (HPA) axis, as well as mitochondrial and synaptic activities, leading to depression, anxiety, cognitive deficits, and post-traumatic stress disorder (PTSD). Interestingly, people with obesity also have a decreased brain volume and are at higher risk of dementia [25]. Importantly, several types of cancer are directly related to obesity [26,27]. Figure 1 shows some of the biological mechanisms through which obesity can lead to cognitive decline, various psychological disorders, or neuropsychiatric manifestations.

Over the last two decades, the correlation of obesity with increased cancer incidence and death has been well established. The estimated percentage of cancer cases caused by obesity alone is 20% [28]. Obesity has also accounted for a significant percentage of deaths in both males and females [28]. The risk of malignancies is influenced by weight change, diet, body fat distribution, and overall physical activity [29]. To demonstrate the characteristic features of cancers (e.g., uncontrolled proliferations), the normal cells must undergo a neoplastic transformation. Obesity causes the chronic activation of cellular growth factor signaling pathways due to excess nutrients, leading to an increased risk for neoplastic transformation [30]. Moreover, the proliferation and growth of cells also depend on the nutrients that support the macromolecule synthesis. Excess energy due to excess food intake gets stored in the form of lipids inside the body, skeletal muscles, and organs (e.g., the liver). The increased level of lipids changes the usual metabolic milieu, which generates an environment that transmits signals for excess nutrients to the cells. As a result, the signaling pathways for angiogenesis, proliferation, glucose uptake, and cell growth are activated, dropping the barrier for oncogenic transformation [31,32].

A recent research study by Calle and Thun established a link between being overweight, obesity, and cancer [27], as they found a risk of colorectal cancer in obese adults. Of note, obese postmenopausal women had a 30–50% greater possibility of breast cancer according to breast cancer studies [27]. The evidence of the association between obesity and numerous types of cancer is shown in Table 2.

## 5. Understanding the General Causes of Obesity

The research studies indicate several factors contributing to obesity; however, the primary cause has been related to an imbalance of energy research studies. Energy imbalances mainly occur when the energy consumed via food (or calorie intake) is not equivalent to the energy utilized (or calories spent) via regular bodily functions such as proper food digestion or staying active. Here, we discuss some of the important factors that contribute to obesity [47].

### 5.1. A Sedentary Routine

Many people do not maintain an active lifestyle and spend countless hours in front of computers, televisions, and other digital gadgets. Technology and more advanced facilities have made it easier for people to do less physical work at home and in the workplace. People also prefer driving rather than walking and eating junk foods over cooking homemade meals. Hence, people with a sedentary lifestyle are prone to obesity and health issues such as coronary artery disease, cancer, and diabetes [48].

### 5.2. Family History

The probability of being overweight or obese increases if one or both parents are overweight or obese. Moreover, the food patterns and lifestyle habits of the parents are picked up by their children. As a result, a child born to obese parents who eat high-calorie foods and are physically inactive will most probably grow up obese [49]. On the other hand, if the family members are not obese and follow healthy eating and exercise habits, the chances of the child being overweight or obese are reduced [49].

### 5.3. Medicines

It is well established that atypical antipsychotic medications (clozapine, olanzapine, risperidone, and quetiapine) induce significant weight gain. Antidepressants, including amitriptyline, mirtazapine, and selective serotonin reuptake inhibitors (SSRIs), may also cause weight gain that cannot be attributed primarily to improved depressive symptoms [50]. Likewise, low mood is associated with increased food consumption, leading to lowered metabolic activity [50]. These changes in stabilizers such as lithium, valproic acid, and carbamazepine have demonstrated a similar effect. Importantly, antiepileptic medications (AEDs) such as sodium valproate, pregabalin, vigabatrin, carbamazepine, and gabapentin can cause weight gain [51,52]. Weight gain associated with antiepileptic medicines (AEDs) is a significant issue in the care of epilepsy patients. Moreover, weight gain is a reported side effect of corticosteroid medications [53]. Furthermore, histamine, a neurotransmitter secreted by the posterior hypothalamus, is also involved in body weight regulation [54], as antagonistic histamine stimulation increases appetite and slows body fat breakdown [55].

### 5.4. Smoking Cessation

Quitting smoking extends the life expectancy by decreasing the risk of major diseases, but is often associated with weight gain issues [56]. The most likely cause of this is an increase in appetite and a decrease in energy expenditure [57]. Nicotine or smoking aids in regulating or managing compulsive eating and overeating. One possibility for weight gain is that nicotine’s appetite-suppressing effect gets reversed [58]. Substitution reinforcement may occur when food is substituted for cigarettes [59]. A meta-analysis examination of weight gain in former smokers (quit for around 12 months) reported that weight gain following smoking cessation was more than previously believed. On average, without using nicotine replacement therapy or other medications, some showed 1.1 kg of weight gain in the 1st month, 2.3 kg in the 2nd month, and a continued increase of up to 4.7 kg following 12 months of smoking cessation. Furthermore, 13% of individuals gained over 10 kg upon cessation; interestingly, 16% lost weight after giving up smoking too [60]. Smokers with a particular history of binge eating during smoking cessation gain significantly more weight than non-smokers [61]. Another way of describing this is that sugary and fatty food consumption stimulates reward circuits in the brain, similar to smoking. [62]. Nicotine withdrawal results in an increased reward threshold, which may induce individuals to consume more carbohydrates and sugar-containing snacks [63].

## 6. Development of Tumor Due to Obesity: Underlying the Biological Mechanism

Obesity is defined as the accumulation of an excessive amount of body fat [64]. Several published statistical studies show that obesity-related cancer alone causes around 100,000 deaths per year. [65]. Of note, BMI, i.e., the ratio of weight to height squared, is used to determine the body fat content (square meters). Whereas a BMI of 25–29 kg/m^2^ is regarded as overweight, a BMI of 30 kg/m^2^ or above is considered obese [64]. Since increased adiposity is highly associated with an increased risk of developing or suffering from various cancers, researchers recently proposed the term “adiponcosis,” which is derived from the fusion of the Latin word ‘adiposis’ (excessive body fat accumulation) with the Greek word ‘oncosis’ (the development of tumors) [66].

The association of obesity with a very high risk of metabolic diseases such as insulin resistance, dyslipidemia, and non-alcoholic fatty liver disease (NAFLD) can lead to carcinogenesis. Chronic inflammation has been described as a significant contributing factor in the evolution and progression of such chronic diseases [67]. In obesity, the inflammatory trigger is metabolic and triggered by extra nutrients. Moreover, specific metabolic cells initiate the inflammatory response via specific metabolic signals to disrupt the normal cellular metabolic homeostasis [68].

The visceral adipose tissue is the largest endocrine organ, which produces proinflammatory cytokines (TNF-alpha, IL-6, and IL-17) and growth factors referred to as adipokines (adiponectin, omentin, chemerin), leading to dyslipidemia, insulin resistance, diabetes, cardiovascular disorders, and cancer [69]. Adipokines are aimed explicitly at storing energy as triglycerides within the lipid droplets of the cytoplasm [69]. Excess nutrients stimulate metabolic signaling pathways such as c-Jun N-terminal kinase (JNK), nuclear factor B (NFB), and protein kinase R. When these pathways are activated, lower levels of inflammatory cytokines are produced, leading to low inflammatory reactions [67]. Excess nutrition and obesity also cause hyperplasia and the enlargement of white adipose tissue adipocytes, significant tissue remodeling, and a rise in free fatty acids, all of which result in adipokine synthesis alterations and low-grade inflammatory responses [70].

**Figure 2:**
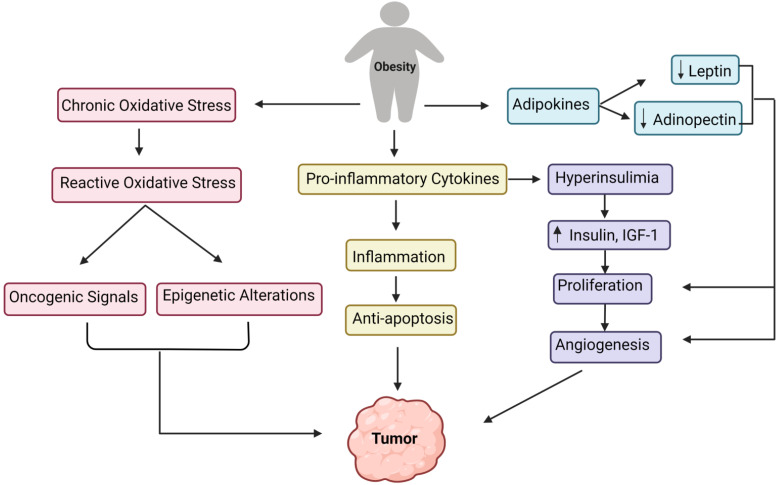
Converging pathways of the underlying mechanisms involved in the tumor development due to obesity.

Moreover, obesity results in increased endoplasmic reticulum stress, which activates the unfolded protein response, activating NF-kB, JNK, and oxidative stress, leading to the overexpression of proinflammatory cytokines [71]. Interestingly, macrophages in visceral adipose tissue have a unique function in significantly increased obesity. They are involved with adipose tissue inflammation and the production of inflammatory cytokines (TNF alpha, IL-6, IL-8, IL-17, IL-18, MCP-1), as well as additional adipokines (resistin, visfatin, and retinol-binding protein 4) [71]. All of these pathways direct to the onset of obesity-related inflammation. While inflammation linked with obesity is predominantly focused on the white adipose tissue, several other tissue types, such as the liver, pancreas, and brain, have also exhibited elevated inflammation under obesity [72]. Figure 2 outlines the various underlying primary mechanisms involved in tumor formation due to obesity.

## 7. Deciphering the Association of Obesity with the Development of GBM

In the case of glioma treatments for obese patients, the two crucial factors are metabolic disorder and the inflammatory milieu. The increased levels of various molecules such as adenosine, adenosine receptors, adenosine kinase, insulin, and lipids, along with the immune response in the case of obese patients, aid in the failure of multimodal therapies for gliomas [73]. The adipocytes that play a vital part in developing obesity, type-2 diabetes, and metabolic syndrome [73] are differentiated from adipose-derived stem cells (ADSCs) [74]. Akimoto et al. investigated the anticancer therapeutic ability of the mesenchymal stem cells (MSCs) by extracting human MSCs from umbilical cord blood (UCB-MSCs) and adipose tissues (AT-MSCs). The MSCs of various origins were cocultured with primary GBM cells to determine their ability to prevent the growth of GBM. The results demonstrated that whereas the UCB-MSCs reduced the growth of GBM and induced apoptosis, the AT-MSCs enhanced the growth of GBM. Interestingly, the AT-MSCs expressed higher quantities of angiogenic factors (vascular endothelial growth factor, angiopoietin 1, platelet-derived growth factor, and insulin-like growth factor) and stromal-derived factor-1 (SDF-1/CXCL12). Hence, the AT-MSCs and GBM when co-transplanted caused highly vascularized tumors. AT-MSCs probably act on different mechanisms such as angiogenesis promotion and apoptosis inhibition towards the formation of GBM in vivo [75]. The genetic association between GBM and obesity has been published by Chen et al., where the fat mass and obesity-associated (FTO) gene on chromosome 16q12.2 was linked with obesity and BMI. The recent interest in the FTO gene and its expression product was piqued by the discovery of FTO as an N6-methyladenosine (m6A) demethylase. FTO predominantly affects the downstream targets’ m6A levels via their 3′ untranslated regions [76]. The oncogenic potential of FTO has also been demonstrated in leukemia and glioblastoma, both of which have high levels of FTO expression [77]. Additionally, it has been established that abnormal insulin signaling accelerates glioblastoma growth, and inhibiting this pathway may provide an alternative therapy to the existing standard care [78]. Since the 1980s, critical molecular participants in this signaling have been identified and intensively explored experimentally [79]. Insulin-like growth factor 1 (IGF1, and the insulin-like growth factor 1 receptor (IGF1R) are required for appropriate brain growth during fetal and postnatal development [80]. Brain cancer cells develop malignant phenotypes in the same processes [81].

Epidemiological studies have suggested an increased risk of cancer in people with T2DM and obesity, primarily mediated by hyperinsulinemia induced by insulin resistance [82]. Hyperinsulinemia promotes the production of IGF-1. The higher insulin and IGF-1 levels have been linked to tumorigenesis in vitro, animal models, and human epidemiological investigations [82]. Growth hormone receptor (GHR) signaling is augmented in hyperinsulinemic conditions due to increased insulin levels in the portal circulation, which results in increased hepatic IGF1 synthesis [83]. In extensive prospective studies and meta-analyses, elevated IGF1 levels have been implicated in developing colorectal, premenopausal breast, and prostate cancer [84]. Additionally, IGF1 induces the tumor’s metastatic spread, possibly by relocating the integrins to the verge of migrating cells and via lamellipodial extension, as expressed in neuroblastoma cells [85]. Thus, hyperinsulinemia can increase circulating IGF1 levels, which can promote tumor growth and metastasis. Experimentally, it has been demonstrated that prostate cancer cells in Noble rats with hormone-sensitive prostate cancer upregulate their intrinsic production of IGF1, implying that the malignant cells rely on circulating IGF1 levels and may also be capable of regulating their growth via IGF1 production [86].

Studies have reported that IGFIR hyperactivation and subsequent signaling results in malignant cell proliferation, motility, metastasis, and antiapoptotic signaling [87,88]. As a result, scientists have targeted IGFIR to inhibit the formation of glioblastoma in vitro and in animal models; IGFIR inhibition successfully inhibited glioblastoma aggregation development [89]. Additionally, metabolic problems such as obesity are related to adverse outcomes in patients with type 2 diabetes and GBM. These metabolic difficulties may substantially affect tumors through various pathways, including the activation of the insulin receptor (INSR) and the closely related insulin-like growth factor 1 receptor (IGF1R) in malignant cells. The investigation demonstrated that INSR was often expressed in GBM surgical tissues and xenograft tumor lines, with mitogenic isoform-A being the most abundant. Insulin at physiologically appropriate doses increased the proliferation and survival of GBM cells, possibly via Akt (protein kinase B) activation [90]. The study conducted by the researchers states that lipid droplets (LDs), which are subcellular organelles that serve as the primary storage locations for neutral lipids, specifically triglycerides (TG) and cholesteryl esters (CE), constitute the lipid core, surrounded by a monolayer of phospholipids [91]. It has been demonstrated that the dysregulation of LD metabolism is associated with a variety of metabolic disorders, including obesity, fatty liver, and atherosclerosis [92].

Moreover, LDs are also detected in various tumor tissues of patients [93]. Current advancements in the interpretation of cancer biology have revealed a new hallmark of malignancies—metabolic reprogramming [94]. Guo et al. were the first to demonstrate that patients with GBM have their lipid metabolism rewired to promote tumor growth [95]. It was discovered that tumor tissues from GBM patients contain high concentrations of LDs utilizing electron microscopy and fluorescence imaging techniques [96]. To avoid lipotoxicity and ER (endoplasmic reticulum) stress, GBM cells may store excess fatty acids and cholesterol in LDs. This research demonstrated that LDs are detected in GBM but not in normal brain tissues or low-grade gliomas, indicating that LDs may serve as potential diagnostic biomarkers for GBM [96].

Interestingly, this was shown in tumor tissues from a large cohort of GBM patients. Furthermore, it has been well established that a higher incidence of LDs in tumor tissues is negatively connected with overall survival, implying that LDs may play a role in GBM progression [96].

## 8. Causes of Childhood Obesity: A Better Understanding of the Associated Concerns of Fatigue, Health Disorders, Cancer, and Death Risks in Children

Obese children have difficulty breathing, a tendency for high blood pressure, early signs of cardiovascular disease, and insulin resistance, in addition to fatigue and fracture risks [97]. Obesity in childhood has also been related to an increased possibility of adult obesity, early death, and disability. Unfortunately, obesity in children has increased drastically in recent decades, making it one of the most common general health problems. The etiology of excessive weight gain is complex, with interactions between genetic, environmental, and biological variables [98]. Hence, it is important to know the causes of childhood obesity, as outlined below.

### 8.1. Genetic Factors and Risk of Childhood Obesity

Heritable factors account for 30% to 50% of adiposity variation [99]. Early-onset obesity and physical assessment symptoms such as short stature, dysmorphic characteristics, pauses in development or intellectual disability (mental retardation), retinal abnormalities, or deafness are common in children with obesity-related genetic disorders. Hypotonia and eating difficulties are common in children with Prader–Willi syndrome during infancy (sometimes accompanied by growth failure), followed by hyperphagia and obesity. The most prevalent single gene defect in children with obesity is mutations in the melanocortin 4 receptor. [100]. Gene mutations in leptin, leptin receptor, proopiomelanocortin, and proprotein convertase are other common defects. Leptin and leptin receptor gene mutations are uncommon. Only rare leptin or leptin receptor mutations have been recorded, most of which are from consanguineous families [101]. The involvement of epigenetic variables in obesity is also becoming clearer. These epigenetic variables could influence how the environment, microbiota, and nutrition combine to promote weight gain [102].

### 8.2. Endocrine Disorder and Childhood Obesity

Only about 1% of children and adolescents with obesity have endocrine factors for their weight gain. Poor linear development, small height, and hypogonadism are common in children with endocrine abnormalities that cause weight gain [103]. Endogenous or exogenous glucocorticoid excess (the use of corticosteroid medication or Cushing syndrome), hypothyroidism, growth hormone insufficiency, and pseudohypoparathyroidism type 1a (Albright hereditary osteodystrophy) are all endocrine illnesses that cause weight gain [103]. Some of the endocrine disorders related to weight gain in children are listed below.

#### 8.2.1. Hypothyroidism

Clinical hypothyroidism with high TSH and low T4 levels is frequently accompanied by some weight gain and a modest increase in BMI (1–2 kg/m^2^). Reduced resting energy expenditure, fluid retention, and slowed linear growth are suggested to be the causes of BMI increases. Subclinical hypothyroidism is seen in approximately 10% of overweight or obese youngsters, although it is a symptom rather than a reason for obesity and does not necessitate thyroxine treatment [104].

#### 8.2.2. Cushing Syndrome or Hypercortisolism

Cushing syndrome occurs due to hypercortisolism, and an exogenous Cushing syndrome type is found in children. Adrenal tumors or hyperplasia in children under 6 years and pituitary microadenoma in older children are the most common causes. Exogenous iatrogenic hypercortisolism (as seen in autoimmune, dermatological, pulmonary, or neoplastic diseases) or even the off-label, over-the-counter use of steroids for treatment for mild respiratory illnesses is also not uncommon [105]. Hypercortisolism is connected to developmental delays, adiposity, hirsutism, increased appetite, and hypertension. Short stature (typically fewer than 3 standard deviations in height), sluggish growth, and mild truncal obesity are all symptoms of growth hormone deficit (GHD) and obesity caused by the hypothalamus [105].

#### 8.2.3. Hypothalamic Obesity

The ventromedial, arcuate, paraventricular, and dorsomedial nuclei of the hypothalamus generate neuropeptides that regulate hunger and energy expenditure. A congenital abnormality or injury causes hypothalamic obesity in the hypothalamus, which causes the nuclei to be disrupted. The primary cause of hypothalamic obesity in children is a surgically treated craniopharyngioma. Pituitary tumors, aneurysms, inflammatory and infiltrative disorders, trauma, cranial irradiation, and surgery are other reasons. Patients that are affected are often tired and have low energy expenditure. GHD, hypothyroidism, precocious or hindered puberty, and diabetes insipidus are all possible endocrinopathies [106]

#### 8.2.4. Rapid Onset Obesity

Hypothalamic dysfunction, hypoventilation, autonomic dysregulation, and neuroendocrine tumors (ROHHADNET) syndrome: This syndromic etiology of pediatric obesity displays weight gain and slowed considerably linear growth, as well as indications of autonomic dysfunction and hypoventilation, between the ages of 2 and 4. It has been linked to various degrees of hypothalamic–pituitary axis involvement, including GH deficit with low IGF1 in some cases, glucocorticoid insufficiency or excess, hypogonadotropic hypogonadism, hyperprolactinemia, hypothyroidism, irregularities in water and sodium homeostasis, adrenal tumors, and so on [107].

### 8.3. Parent–Child Interactions: A Risk Factor for Childhood Obesity

The human brain evolved in a social context [108], and the complex physiologic processes maintaining energy balance are intimately linked to the autonomic nerve system, which develops quickly in childhood [109]. The emotional quality of parent–child connections can raise a child’s risk of obesity in a variety of ways, and identifying these pathways is an important study area [108]. The research shows that loving and warmth-related parenting can alter the autonomic nerve system’s function in children to maintain a healthy weight [110].

The sympathetic nervous system and the hypothalamic–pituitary–adrenal (HPA) axis transmit neuroendocrine changes in response to stress, causing physiological arousal and influencing appetite and mood [110]. The degree to which the brain’s messages and the body’s response are in synchronization may aid in determining whether a person’s appetite rises or falls in response to stress [111]. Long-term or severe stress can cause habituation and insufficiency in the brain’s energy demand signaling, necessitating more food consumption to maintain glucose homeostasis [112]. Another possible link between the nature of the parent–child relationship and obesity is the capacity of children for self-regulation. Self-regulation is a multifaceted concept that spans the conscious and unconscious domains [113]. Additionally, a study assessed children’s self-regulating behavioral capacities between the ages of three and five. It concluded that self-regulation failure in early childhood might contribute to an excessive weight gain of a child during early adolescence [114].

In the United States and other countries, child obesity has become one of the most critical public health issues [115]. Multiple major obesity-related comorbidities have emerged due to the rising prevalence of childhood obesity [116]. The rates of obesity differ by ethnicity, race, and socioeconomic situation [117]. Low-income people are also more likely to be obese [118]. Obesity in children is caused by complex interactions between several environmental, genetic, and ecological forces.

## 9. Assessing the Risks of GBM with Childhood Obesity

Children: The statistical data show that by 2014, up to 1.9 billion people (18 years and older) were overweight (39%) and over 600 million (13%) were obese. Surprisingly, 42 million children aged five and younger were found to be overweight or obese in 2014 alone. The World Health Organization (WHO) estimates that by the year 2025, 1 out of 5 people will be obese, raising major public health concerns, even in children [119]. Importantly, obese children experience breathing difficulties; early indicators of insulin resistance, neuropsychological issues; and higher risks for hypertension, cancer, and cardiovascular disease [119,120]. Since childhood obesity is highly connected with a higher likelihood of adult obesity, the risks of developing cancer and threats of early mortality in adulthood become high for them. Henceforth, it is important to comprehend the causes of obesity and its relevance in children.

Childhood central nervous system (CNS) tumors are the second most frequently occurring and persistent type of solid tumor. In the United States, the incidence rate of CNS tumors in children and adolescents is around 5.67 per 100,000 person-years [121]. The signs and symptoms vary according to several factors, including the tumor’s location, the child’s age, and the rate at which the tumor progresses. Supratentorial tumors are more prevalent in newborns and children up to the age of 3 and after the age of 10, while infratentorial tumors are more commonly seen in children between 4 and 10 years of age [122]. The clinical manifestations of the growth of tumors in infants are relatively unspecific, such as microcephaly, developmental delay, irritability, and vomiting [123], whereas the typical clinical presentation in older children involves localized neurologic impairments and symptoms associated with increased intracranial pressure caused by a restriction of the normal flow of cerebral spinal fluid (CSF), resulting in hydrocephalus. The non-localizing symptoms associated with prolonged intracranial pressure involve nausea, headaches, cranial nerve deficits, and vomiting. [124]. Children develop high-grade gliomas (HGGs) at an annual rate of 0.8 per 100,000 children [124]. HGGs account for approximately 20% of all pediatric gliomas and include anaplastic astrocytoma (AA), diffuse intrinsic pontine glioma (DIPG), and GBM [125].

Children, like adults, have a dismal prognosis for malignant (high-grade) gliomas such as anaplastic astrocytomas (WHO grade III) and GBM [126]. Pediatric HGGs are histologically identical to adult HGGs but are molecularly distinct entities, making clinical data from adults nearly impossible to translate to pediatrics. While approximately half of all adult GBMs show MGMT (methyl guanine ethyl transferase) methylation, impairing DNA repair, the importance of MGMT methylation in pediatric patients is less apparent [127]. It has been estimated that children diagnosed with malignant brain tumors have a survival rate of approximately 70%. These children have an increased risk of dyslipidemia, obesity, and insulin resistance [128]. Howell et al. used Mendelian randomization (MR) and genetic variants to uncover evidence of a link between risk factors and the development of glioma. They discovered that genetically predicted severe childhood obesity raises the chance of all gliomas and GBM [129]. Interestingly, the research has also indicated opposite associations, as survivors of childhood brain tumors (SCBT) show risks of overweight and obesity due to pain; medicinal side effects; and decreased physical activity, mobility, coordination, sleep, sadness, and pituitary hormone functions [130].

**Figure 3:**
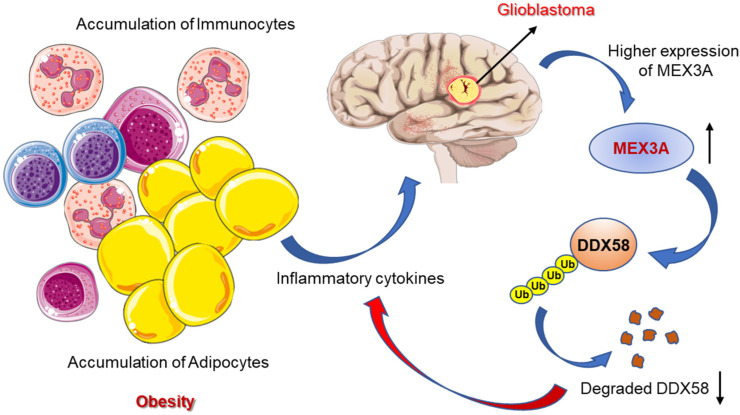
The release of inflammatory cytokines contributing to GBM is regulated by the MEX3A, which acts on DDX58 and causes ubiquitin-mediated degradation.

GBM research requires the identification and characterization of newer and specific molecular players. A recent study identified the RNA-binding ubiquitin ligase MEX3A from a gene expression analysis of a publicly available dataset and tried assessing its functional role in GB tumorigenesis. Interestingly, they found significantly upregulated expression of MEX3A in GB specimens along with a very low level of RIG-I (a tumor suppressor protein involved in apoptosis and immune response) [131]. The study demonstrated a correlation between the two molecules, as MEX3A can bind to RIG-I to induce the ubiquitylation and proteasome-dependent degradation of RIG-I [132]. Moreover, the functional loss the of the MEX3A gene suppresses GB cell growth by increasing the RIG-I protein levels (Figure 3). The findings could be of special interest for childhood-obesity-induced GBM, as targeting MEX3A as a novel molecular approach can regulate the cytokine production from the adipocytes and immunocytes to prevent the development or progression of GBM in children [133].

## 10. Conclusions

The pathophysiology of excessive weight gain or obesity is complex, as it involves several interactions between genetic, environmental, and biological components for an individual. Interestingly, the convergent pathways involving various adipokines, inflammatory molecules, and insulin-resistance-associated obesity with cancer have been brought to the forefront. Moreover, increased adiposity has been linked to a higher chance of acquiring or suffering from various cancers, including GBM and the aggressive forms of tumor originating in the CNS. GBM can strike anyone at any age, but a Mendelian randomization (MR) and genetic variant study indicated that obese children have a plausible chance for developing all gliomas, including GBM.

Unfortunately, obesity in children has escalated exponentially, making it one of the most predominant public health problems. Obese children suffering from GBM demonstrate several health issues along with neuropsychiatric manifestations and an increased risk of death. Moreover, the survival rate of obese children diagnosed with dyslipidemia, obesity, and insulin resistance decreases with the development of malignant brain tumors.

Interestingly, now we know that the C subtype of GBM is associated with the amplification of EGFR and EGFR vIII mutations, and the proneural subtype is also associated with a younger age at diagnosis, along with IDH1/2 and TP53 mutations. On the other hand, the M subtype involves more frequent NF1 mutations and the enrichment of gene signatures associated with EMT (epithelial-to-mesenchymal transition). Personalized treatments for childhood-obesity-induced cancers can be used to study the fine details of the various GBM subtypes. Moreover, therapeutic interventions to prevent the adverse effects of obesity on cancer can have several other potential targets. It has also been seen that patients on insulin or insulin-secreting agents (e.g., sulphonylureas) are at a higher risk of developing cancer compared to patients on oral insulin-sensitizing agents, e.g., metformin or thiazolidinediones (TZDs). This is one of most important pending research questions, which needs special attention and further investigation for appropriate drug discovery. Another potential therapeutic approach in cases of obesity-induced cancer progression is the chemo-preventive modulation of the inflammatory pathways. Interestingly, targeting adipokines for weight loss and bariatric surgery has reduced the incidence and metastasis of cancer. In contrast, a study examined the effects of obesity-related factors on glioma risk using genetic markers in a MR framework that eliminates confounding variables and is unaffected by reverse causality [134]. The study revealed no indication that obesity-related factors contribute to the development of glioma [134]. However, the positive correlation between obesity and glioma far outweighed the negative correlation. Additionally, obesity is known to accelerate other degenerative conditions and contributes to aging [135], which can also be controlled [136]. Out of the known molecular drivers that play a role in obesity-induced GBM, the RIG-I protein and MEX3A could be of potential interest, as they can regulate the cytokines released from the adipocytes and inflammatory cells of an obese person and can regulate the occurrence or development of GBM.

Further, research and medical studies are needed to determine the precise underlying mechanisms that communicate among themselves and link GBM with childhood obesity. More focused studies in different populations as well as in physiological conditions are needed in order to eradicate the childhood GBM cases induced by obesity or abnormally high BMI. This will contribute significantly to the overall growth of a nation [137,138] in the terms of both the economy and public health.

## Authors Contributions

P.S., S.G. (Shampa Ghosh) and J.K.S. conceptualized the content of the paper. All authors contributed to all versions of the manuscript and the illustrations. All authors have read and agreed to the published version of the manuscript.

## Figures and Tables

**Table 1:** Neuropsychiatric symptoms associated with the locations of GBM tumors in patients.

S. No.	Patient’s Age	Patient’s Gender	Location of GBM Tumor	Neuropsychiatric Symptoms	Reference
1.	37 years	Male	Left frontal lobe	Headache, anomic aphasia, paraphasia, and dysgraphia	[17]
2.	34 years	Male	Right frontal lobe	Delirium, depression	[18]
3.	39 years	Male	Frontal bilateral lobes	Aphasia, headache, anxiety, PTSD, left side numbness	[19]
4.	63 years	Male	Right temporal lobe	Partial complex seizure	[20]
5.	59 years	Male	Left temporal lobe	Slurred speech, memory impairment, clonic movement of arms and legs, weakness, sharp pain in limbs, and rapid clonic tremor	[21]
6.	55 years	Female	Left frontoparietal lobes	Panic attacks, agoraphobia. Depression and Right-sided weakness	[22]
7.	63 years	Female	Medulla oblongata	Gait disturbances, dizziness, loss of appetite	[23]
8.	29 years	Female	Left thalamus	Memory impairment, borderline personality disorder, depressive symptoms, PTSD	[24]

**Table 2:** This shows the link between obesity and the development of various types of cancer.

S. No.	Cancer Type	Association with Obesity	Reference
1.	Prostate Cancer	Obesity promotes low-grade inflammation linked to prostate cancer progression by compromising treatment and diagnosis.	[33]
2.	Breast Cancer	In obese women, aromatization activity due to elevated levels of estrogen, the overexpression of insulin resistance, proinflammatory cytokines, IGFs, oxidative stress, and hypercholesterolemia contributes to the development of breast cancer.	[34]
3.	Lung Cancer	Abdominal obesity has a significant role in the development of lung cancer. Smoking is one of the leading causes of the development of lung cancer; people who smoke regularly have an increased BMI. The reduced levels of sex-hormone-binding globulin and elevated estrogens and androgens are associated with obesity and an increased risk of lung cancer. However, the exact mechanism is not understood.	[35]
4.	Bladder cancer	Obesity is a potential risk factor for recurrence, progression, or death with bladder cancer.	[36]
5.	Colorectal Cancer	Visceral and abdominal fat increase the risks of colorectal cancer by up to 30–70% in men and are linked with worse outcomes and recurrence.	[37]
6.	Kidney Cancer	Renal cell cancer (RCC) is the main form of kidney cancer. Studies have reported that people with a high waist-to-hip ratio (WHR), waist circumference (WC), and increased BMI are risk factors associated with RCC.	[38]
7.	Hodgkin Lymphoma (HL)	It has been elucidated that inflammation is common both in HL and obesity. The interaction of molecules released by adipocytes and the tumor microenvironment associates obesity with an increased risk of developing HL.	[39]
8.	Melanoma	Adipocytes provide nutrients to melanoma cells. Adipokines released by adipocytes stimulate the progression of myeloma cells. Moreover, it has been reported that insulin resistance and hyperinsulinemia may promote the growth of myeloma.	[40]
9.	Pancreatic Cancer	Obesity increases the risk of pancreatic cancer through mechanisms that are not fully understood. Inflammation and hormone imbalance could be plausible causes. Excess abdominal adiposity is one of the few controllable risk factors for developing pancreatic cancer.	[41]
10.	Thyroid Cancer	Adiponectin (APN) is one of the vitally essential adipocytokines. In obese individuals, there are reduced levels of APN. Similarly, reduced levels of APN have been found in patients with thyroid cancer and metabolic syndrome.	[42]
11.	Endometrial Cancer	Visfatin, leptin, and resistin are associated with endometrial cancer proliferation, growth metastasis, and invasion.	[43]
12.	Bone Cancer	Bone marrow adipocytes (BMAds) increase in size and number during obesity and can initiate bone cancer or cancer within the bone marrow. The BMAds provide nutrients to tumor cells and help in tumor cell proliferation.	[44]
13.	Gastric Cancer	Obesity is associated with the occurrence of gastric cancer. The mechanism involved includes insulin resistance; higher levels of IGFs; and altered leptin, ghrelin, and adiponectin levels.	[45]
14.	Neuroblastoma	Lim et al. reported on a 29-month-old Korean female who developed neuroblastoma and showed clinical features of ROHHAD. The laboratory examinations revealed high levels of IGF-1, prolactin, sex hormone, cortisol, and lactate dehydrogenase.	[46]

## Data Availability

Not applicable.

## Abbreviations

GBM	Glioblastoma multiforme
CNS	Central nervous system
BTs	Brain tumors
TCGA	The Cancer Genome Atlas
NSAIDs	Non-steroidal anti-inflammatory drugs
BMI	Body mass index
ASCVD	Atherosclerotic cardiovascular disease
SSRIs	Selective serotonin reuptake inhibitors
PTSD	Post-traumatic stress disorder
JNK	Jun N-terminal kinase
NFB	Nuclear factor B
MCP-1	Monocyte chemoattractant protein-1
TNF	Tumor necrosis factor
APN	Adiponectin
BMAds	Bone marrow adipocytes
RCC	Renal cell cancer
WC	Waist circumference
HL	Hodgkin lymphoma
HPA	Hypothalamic–pituitary–adrenal
MSCs	Mesenchymal stem cells
UCB	Umbilical cord blood
FTO	Fat mass and obesity-associated
m^6^A	N6-methyladenosine
IGF-1	Insulin-like growth factor 1
IGFIR	Insulin-like growth factor 1 receptor
LDs	Lipid droplets
TG	triglycerides
CE	Cholesteryl esters
ER	Endoplasmic reticulum
DIPG	Diffuse intrinsic pontine glioma
SCBT	Survivors of childhood brain tumors
MR	Mendelian randomization
